# Estimation of passive and active properties in the human heart using 3D tagged MRI

**DOI:** 10.1007/s10237-015-0748-z

**Published:** 2015-11-26

**Authors:** Liya Asner, Myrianthi Hadjicharalambous, Radomir Chabiniok, Devis Peresutti, Eva Sammut, James Wong, Gerald Carr-White, Philip Chowienczyk, Jack Lee, Andrew King, Nicolas Smith, Reza Razavi, David Nordsletten

**Affiliations:** 1 Division of Imaging Sciences and Biomedical Engineering, St Thomas’ Hospital, King’s College London, 4th Floor, Lambeth Wing, London, SE1 7EH UK; 2 Inria Saclay Ile-de-France, MΞDISIM Team, Palaiseau, France; 3 Department of Cardiology, Guy’s and St Thomas’ NHS Foundation Trust, London, SE1 7EH UK; 4 Department of Clinical Pharmacology, Guy’s and St Thomas’ NHS Foundation Trust, London, UK; 5 Faculty of Engineering, University of Auckland, Auckland, New Zealand

**Keywords:** Cardiac mechanics, Parameter estimation, 3D tagged MRI, Patient-specific modelling

## Abstract

Advances in medical imaging and image processing are paving the way for personalised cardiac biomechanical modelling. Models provide the capacity to relate kinematics to dynamics and—through patient-specific modelling—derived material parameters to underlying cardiac muscle pathologies. However, for clinical utility to be achieved, model-based analyses mandate robust model selection and parameterisation. In this paper, we introduce a patient-specific biomechanical model for the left ventricle aiming to balance model fidelity with parameter identifiability. Using non-invasive data and common clinical surrogates, we illustrate unique identifiability of passive and active parameters over the full cardiac cycle. Identifiability and accuracy of the estimates in the presence of controlled noise are verified with a number of in silico datasets. Unique parametrisation is then obtained for three datasets acquired in vivo. The model predictions show good agreement with the data extracted from the images providing a pipeline for personalised biomechanical analysis.

## Introduction

Integrated imaging and mathematical modelling hold significant potential for augmenting and improving current clinical practice. The ongoing advancement in computational modelling enables fast and efficient simulation of complex physiological systems (Casoni et al. 2014; Gurev et al. 2015; Lafortune et al. 2012). Biomechanical models provide the distinct advantage in that they enable clinicians and researchers to estimate characteristics of the heart that are otherwise difficult or impossible to measure in vivo, such as tissue stiffness and contractility, local strains and stresses. These model-derived metrics yield additional, potentially more sensitive, indicators of health and disease. Consequently, personalised cardiac modelling has generated significant clinical interest (Ge and Ratcliffe 2009; Sermesant et al. 2012; Yacoub and Terracciano 2011), actively stimulating research into model developments (Chabiniok et al. 2012; Krishnamurthy et al. 2013; Marchesseau et al. 2013; Neal and Kerckhoffs 2010) that may improve diagnosis, facilitate prognosis and assist in treatment planning.

Advances in medical imaging and image processing are paving the way for the use of cardiac biomechanical modelling in clinical applications. Magnetic resonance imaging (MRI), computed tomography and echocardiography have advanced to the state where anatomy, motion (kinematics) and flow can all be routinely imaged in patients. Various types of cardiac MR sequences, such as high-resolution three-dimensional (3D) anatomy at multiple cardiac states (Uribe et al. 2008), cine images of cardiac motion (Uribe et al. 2007; Usman et al. 2013, 2015), 4D phase contrast MRI (Markl et al. 2011) and 3D tagged MRI (Rutz et al. 2008), provide an extensive set of data for modelling. Motion tracking in 3D tagged images (Chandrashekara et al. 2004) in particular enables the extraction of three-dimensional displacements of individual points in the myocardium and can therefore serve as basis for quantification of the mechanical properties of cardiac tissue (Mojsejenko et al. 2014; Xi et al. 2013). Moreover, non-invasive approaches to estimating pressure from imaging data (Buyens et al. 2005; Donati et al. 2015; Ebbers et al. 2001; Tyszka et al. 2000) or peripheral cuff measurements (Brett et al. 2012; Chen et al. 1997) are becoming more advanced and pervasive in clinical assessments.

Leveraging this quantitative data for construction of cardiac mechanics models, it is essential to provide reliable and robust models and parameterisation pipelines for processing patient data to producing results that enable medical interpretation. In this context, model selection is of critical import, requiring a balance between the objectives of the analysis and input extractable from the data. This mandates selection of a model that is inherently a compromise, providing sufficient richness to represent the physiological processes of interest (i.e. sufficient model fidelity), while limiting the number of patient-specific parameters to ensure identifiability. Many modelling approaches have been reported in the literature (Camara et al. 2015), using a variety of laws to describe the passive (Costa et al. 2001; Guccione et al. 1995; Holzapfel and Ogden 2009) and active (Chapelle et al. 2012; Kerckhoffs et al. 2003; Niederer et al. 2011) behaviour of the myocardium. Quantification of mechanical properties from imaging data has also been pursued (Augenstein et al. 2005; Chabiniok et al. 2012; Chandrashekara et al. 2004; Gao et al. 2015; Göktepe et al. 2011; Imperiale et al. 2011; Mojsejenko et al. 2014). However, model complexity often limits the ability to identify patient-specific parameter values uniquely (Hadjicharalambous et al. 2014b; Xi et al. 2013), thus adding uncertainty to the quantitative characterisation the model can provide.

The aim of this work is to develop a relatively simple model characterising full-cycle left-ventricular (LV) mechanics with sufficient accuracy, and an associated pipeline for reliable estimation of a limited number of patient-specific parameters, based on a non-invasively acquired dataset including 3D tagged MRI. Successful implementation of this pipeline would provide a basis for linking the model-derived values, such as passive and active parameters, to the underlying pathophysiology. The computational model and the associated parametrisation protocol introduced here are building on our previous work for passive parameter estimation from 3D tagged data (Hadjicharalambous et al. 2014b) and full-cycle parameter estimation from pressure–volume data (Asner et al. 2015). A reduction in the Holzapfel–Ogden material model (Holzapfel and Ogden 2009) is used that was shown to balance identifiability and model fidelity across a range of passive behaviours (Hadjicharalambous et al. 2014b). Novel external boundary conditions are applied, so that deformations are driven by LV volumes and basal long-axis motion extracted from processed image data. Finally, a simplified active constitutive law is integrated into the model, providing an estimate of the active tension in the myocardium over the cardiac cycle.

In passive parameter estimation, optimal values are selected based on the $L_2(\varOmega _0)$-norm difference between 3D motion fields extracted from the data and model predictions. Active tension through the cardiac cycle is estimated based on a combined functional incorporating relative $L_2$-norm displacement errors and cavity pressure errors.

Parameter identifiability and model sensitivity are verified in a series of tests based on in silico data emulating acquired data and processing errors. These tests provide a useful tool for evaluating the estimation pipeline under perfect or near-perfect model fidelity and allow us to assess the errors in the estimated quantities. With the view of using the process in patient-specific modelling, we then apply the model and the parameterisation protocol to three in vivo datasets including MR images and pressure data as an important verification step. Model fidelity is reduced for in vivo data, and the feasibility of the estimation process, as well as the reliability of computed parameter values, needs to be re-evaluated for potential use in clinical cases. Parameter identifiability is verified in the in vivo cases, and good agreement between the data and the model can be observed.

Section 2 gives the details of the full-cycle LV mechanics model used in the pipeline and describes the datasets and the process for model personalisation. The results for all of the in silico and the in vivo tests are presented in Sect. 3. The outcomes of the testing, along with limitations and future work, are discussed in Sect. 4.

## Methods

The specifics of the proposed pipeline for personalisation of full-cycle LV mechanics models are described below. First, we present the cardiac mechanics model, including the governing equations, the passive and active constitutive laws, and the boundary conditions. A minimal clinical dataset required is outlined next, and the procedure for personalisation of the model based on this dataset is described. We also present the steps involved in generating the in silico datasets.

### Cardiac mechanics model

#### Governing equations

The equations used to model myocardial deformation over the cardiac cycle are derived from the basic conservation principles (Bonet and Wood 2008). We employ a standard Galerkin finite element formulation of the solid mechanics problem, while choosing specific constitutive laws and boundary conditions to ensure good model fidelity and parameter identifiability based on the available data. The details of the model are described below.

Let $\varOmega _0 \in {\mathbb {R}}^3$ be the reference configuration of the domain of interest (the unloaded myocardium) with ${\varvec{X}}$ denoting the corresponding reference coordinate. Let also $\varOmega _t \in {\mathbb {R}}^3$ be the deformed domain with ${\varvec{x}}$ the corresponding physical coordinate at a given time $t>0$. The mapping between the deformed and the reference coordinates is assumed to be diffeomorphic at all times. At any time $t>0$, the displacements of material points ${\varvec{u}}={\varvec{x}}-{\varvec{X}}$ and the hydrostatic pressures *p* together with the Lagrange multiplier vector ${\varvec{\lambda }}$ (see Sect. 2.1.3) are found as the saddle point of the energy potential functional $\varPi$ (Bonet and Wood 2008):

1 $$\begin{aligned} ({\varvec{u}},p,\varvec{\lambda })(t): \ \varPi ({\varvec{u}},p,\varvec{\lambda }) = \inf _{{\varvec{v}}} \sup _{q,{\varvec{\mu }}} \varPi ({\varvec{v}},q,\varvec{\mu }), \end{aligned}$$

where $({\varvec{u}},p,{\varvec{\lambda }})(t)$ and $({\varvec{v}},q,{\varvec{\mu }})(t) \in X\times Y \times \varLambda$, with $X\times Y\times \varLambda$ a stable combination of Sobolev spaces for any $t\ge 0$ (Brenner and Scott 2008). The solution must coincide with a critical point of the functional:

2 $$\begin{aligned} D_{({\varvec{u}},p,{\varvec{\lambda }})} \varPi ({\varvec{u}},p,{\varvec{\lambda }})[{\varvec{v}},q,{\varvec{\mu }}] = 0 \end{aligned}$$

for any $({\varvec{v}},q,{\varvec{\mu }})(t) \in X\times Y\times \varLambda$ at a given $t>0$. Eq. (2) provides the weak form equations for solving the solid mechanics problem.

#### Constitutive relations

The precise form of the energy functional depends on the choice of models that represent the myocardium. The total energy can be split into internal and external energy terms: $\varPi = \varPi ^{\text {int}} + \varPi ^{\text {ext}}$. The former is determined solely by the properties of the material, and the latter comes from the boundaries where external forces are applied. We assume that myocardial tissue behaves like an incompressible hyperelastic material, where the internal strain energy can be represented as the sum of the total passive ($W_p$) and active ($W_a$) strain energies of the body and an incompressibility penalty:

3 $$\begin{aligned} \varPi ^{\text {int}}({\varvec{v}},q) = \int _{\varOmega _0} \left\{ W_p({\varvec{v}}) + W_a({\varvec{v}}) + q(J_{{\varvec{v}}}-1) \right\} \, {\mathrm {d}} {\varvec{X}}. \end{aligned}$$

$J_{{\varvec{v}}}=\det ({\varvec{F}}_{{\varvec{v}}})$ is the determinant of the deformation gradient tensor for a deformation ${\varvec{v}}$: ${\varvec{F}}_{{\varvec{v}}} = \nabla _{\varvec{X}} {\varvec{v}}+ {\varvec{I}}$.

The constitutive laws defining the strain energy functions are usually derived from experimental data on material response to different loading conditions. It is established that the passive stress–strain relationship for the myocardium is highly nonlinear (Nash and Hunter 2001), and several laws have been used to model such behaviour (Costa et al. 2001; Guccione et al. 1995; Holzapfel and Ogden 2009). A good combination of model fidelity and parameter identifiability in estimation based on 3D tagged data is observed in the reduced form (Hadjicharalambous et al. 2014b) of the Holzapfel–Ogden law (Holzapfel and Ogden 2009):

4 $$\begin{aligned} W_p({\varvec{v}}) = \frac{a}{2b}\left\{ e^{b(I_{\varvec{C}_v}-3)}-1\right\} +\frac{a_f}{2b_f} \left\{ e^{b_f(I_{\varvec{C}_{{\varvec{v}},f}}-1)^2}-1\right\} , \end{aligned}$$

where $a, a_f, b$ and $b_f$ are constant parameters, $I_{\varvec{C}_{{\varvec{v}}}} = {\varvec{C}}_{{\varvec{v}}} : {\varvec{I}}$ is the first invariant of the right Cauchy-Green deformation tensor $\mathbf C_{{\varvec{v}}} = {\varvec{F}}_{{\varvec{v}}}^T {\varvec{F}}_{{\varvec{v}}}$, and $I_{{\varvec{C}}_{{\varvec{v}},f}} = {\varvec{C}}_{{\varvec{v}}} : ({\varvec{f}} \otimes {\varvec{f}})$ with ${\varvec{f}}$ denoting the myocardial fibre direction at a specific point in the domain. The values of the exponents are not estimated, but instead fixed at $b=b_f=5$, ensuring good agreement of the diastolic pressure–volume curve with the experimentally derived Klotz curve (Hadjicharalambous et al. 2014b; Klotz et al. 2006).

Similarly, a number of constitutive laws can be used to model active behaviour of the myocardium (Chapelle et al. 2012; Kerckhoffs et al. 2003; Niederer et al. 2011). We sought to employ a simple version of these laws that would limit parameter uncertainties. Prescribing a single time-dependent active tension $\alpha (t)$ over the whole myocardium led to non-physiological contractile behaviour of the model, but incorporating the effect of cell length dependence $\phi _{\text {iso}}$, as described in Kerckhoffs et al. (2003), allowed us to resolve the abnormality while keeping the model sufficiently simple:

5 $$\begin{aligned} W_a({\varvec{v}}) = \alpha (t) \phi _{\text {iso}}({\varvec{C}}_{{\varvec{v}},f}). \end{aligned}$$

#### Boundary conditions

Any external loading affecting the system can be incorporated into the model using the Lagrange multiplier method (Babuska 1973), with individual multipliers representing pressures on each of the boundaries. We can prescribe boundary conditions on the endocardium and the base of the left ventricle, so that $\varPi ^{\text {ext}} = \varPi ^{\text {ext}}_{\text {endo}} + \varPi ^{\text {ext}}_{\text {base}}$. The full Lagrange multiplier vector combines the endocardial and the base components: ${\varvec{\lambda }} = (\lambda _{\text {endo}}, \varvec{\lambda }_{\text {base}})$.

The endocardial energy term penalises deviations of the model from given LV cavity volumes:

6 $$\begin{aligned} \varPi ^{\text {ext}}_{\text {endo}} = \lambda _{\text {endo}}\left( V(t)-V^{\text {data}}(t)\right) , \end{aligned}$$

where *V*(*t*) is the volume of the cavity computed from model displacements ${\varvec{v}}$ (Asner et al. 2015), and $V^{\text {data}}(t)$ the volume computed from 3D tagged data, both at a given time *t* in the cycle. While it is common to drive cardiac mechanics models with cavity pressures (Nash and Hunter 2001; Wang et al. 2009; Xi et al. 2013), measuring these pressures non-invasively over the cardiac cycle is currently impossible. At the same time, cavity volumes can be easily derived from the time-resolved images. A volume-driven model can therefore be more easily personalised using routinely acquired patient data. Alternatively, a lumped parameter model can be coupled into the system in order to drive the cardiac cycle (Sainte-Marie et al. 2006; Kerckhoffs et al. 2007; Krishnamurthy et al. 2013). However, any such model requires additional personalisation based on the data that are already available, which is complicated by the 3D–0D coupling.

The base energy term penalises deviations of the model from given displacements at the base of the ventricle:

7 $$\begin{aligned} \varPi ^{\text {ext}}_{\text {base}} = \int _{\varGamma _0^{\text {base}}} \varvec{\lambda }_{\text {base}} \cdot \left( {\varvec{v}}- {\varvec{u}}_{\text {base}} - \frac{1}{2}{\varvec{K}}_b {\varvec{\lambda }}_{\text {base}} \right) \, {\mathrm {d}} {\varvec{X}}, \end{aligned}$$

where ${\varvec{\lambda }}_{\text {base}} \in {\mathbb {R}}^3, {\varvec{u}}_{\text {base}}$ are the deformations extracted from the images, and ${\varvec{K}}_b$ the penalty matrix. By choosing ${\varvec{K}}_b = \varepsilon ({\varvec{I}} - {\varvec{n}}_b(t) \otimes {\varvec{n}}_b(t) )$ with $0 < \varepsilon \ll 1$, where ${\varvec{n}}_b(t)$ is the normal vector to the base plane at time *t*, we enforce strict adherence to ${\varvec{u}}_{\text {base}}$ in the ${\varvec{n}}_b(t)$ direction and relaxed adherence in the base plane controlled by $\varepsilon$. A weak Dirichlet condition on the base can be applied by setting $\varepsilon = 0$.

#### Finite element discretisation

A standard Galerkin finite element method is employed to find a numerical solution to Eq. (2), as described in Asner et al. (2015), Hadjicharalambous et al. (2014a) and Nash and Hunter (2001). The domain is discretised to produce a computational mesh $\varOmega ^h$, and the spaces in the weak form are substituted for their discrete counterparts:

8 $$\begin{aligned} D_{({\varvec{u}}^h,p^h,{\varvec{\lambda }}^h)} \varPi ({\varvec{u}}^h,p^h,\varvec{\lambda }^h)[{\varvec{v}}^h,q^h,{\varvec{\mu }}^h] = 0 \end{aligned}$$

for any $({\varvec{v}}^h,q^h,{\varvec{\mu }}^h)(t) \in X^h\times Y^h\times \varLambda ^h$ at a given $t=t_n, \ n=0,\ldots ,N$ with $0\le t_0<t_1<\cdots <t_N$. The discrete spaces are quadratic for displacements and linear for pressures in all tests to preserve stability in the mixed formulation.

The combination of the governing equations, constitutive relations and boundary conditions fully defines the cardiac mechanics model used in this work, with the finite element method providing a practical tool to solve the model equations. Personalisation of this model relies on a non-invasively acquired dataset, which is described below.

### Non-invasive datasets

For a given set of patient-specific parameter values $a, a_f$ and $\alpha (t)$ the data required for simulating cardiac mechanics using the above model includes the following:

1. reference geometry $$\varOmega _0$$,
2. fibre orientations $${\varvec{f}}$$,
3. LV cavity volumes over the cycle $V^{\text {data}}(t)$,
4. displacements of the LV base plane ${\varvec{u}}_{\text {base}}$. The estimation of $a, a_f$ and $\alpha (t)$ relies on additional information:
5. local displacements throughout the entire LV and
6. LV cavity pressure estimates: a single value or a full-cycle trace, as discussed below.

Obtaining the reference geometry from image data is a non-trivial task, since the myocardium is not observed in its unloaded state during the cardiac cycle. In practice, end-systolic (Wang et al. 2009) and early (Mojsejenko et al. 2014; Xi et al. 2013), or even end-diastolic (Dokos et al. 2002) geometries have been used as reference for simulation purposes. The LV cavity volume at end systole and early diastole is normally close to the refence volume estimates. Moreover, passive parameter estimates were shown to be minimally affected by changing the reference state from end-systolic to early-diastolic geometries in an idealised LV in Hadjicharalambous et al. (2014b). At the same time, the effect of selecting the reference as an early-diastolic motion state, where the myocardium is known to experience residual active tension, needs to be quantified.

An alternative approach to obtaining the reference state is the solution of an inverse mechanics problem (Krishnamurthy et al. 2013; Xi et al. 2014). However, any such model is directly dependent on the values of material parameters estimated in the forward problem, as well as the boundary tractions, and it is not clear that the coupled problem is well posed. We investigated the potential for joint estimation of passive parameters and the reference state in “Appendix 1” and were unable to obtain satisfactory results for the available dataset. The observed lack of identifiability was indicative of strong coupling between the estimated values.

In the present pipeline, the end-systolic geometry was used as the reference state, ensuring consistent selection of the reference state between cases and balancing the accuracy of approximation with resulting parameter identifiability.

A commonly used linear fibre angle distribution between $60^{\circ }$ on the endocardial and $-60^{\circ }$ on the epicardial surface (Spotnitz 2000; Streeter et al. 1969) was used in the model. In practice, it is possible to generate personalised fibres based on diffusion tensor MRI data (Rohmer et al. 2006; Nagler et al. 2013; Stoeck et al. 2014; Toussaint et al. 2013). However, at the moment scan duration as well as spatial resolution and accuracy of the acquired data is still prohibitive for clinical use.

The reference displacements of the computational mesh through the cardiac cycle were obtained by tracking the motion of the myocardium in 3D tagged images. The process is based on a non-rigid registration algorithm (Chandrashekara et al. 2004; Rueckert et al. 1999; Shi et al. 2012, 2013) and carried out with the Image Registration Toolkit^1^ (IRTK). The tracking procedure provides a transformation for each voxel of the image. This can be applied to the vertices of a computational mesh positioned in the physical coordinates, producing the deformed states at the time points in the cardiac cycle where 3D tagged frames were available.

As discussed above, the model is driven by LV cavity volumes, since the corresponding pressure curve cannot be measured non-invasively. The volumes can be computed with reasonable accuracy for each deformed state of the computational mesh, and the pressures $\lambda _{\text {endo}}$ are then computed in the simulations. Due to the linear nature of the chosen parametrisation, scaling all of $a, a_f$ and $\alpha (t)$ by a constant factor does not affect the displacements, and scales the pressures $\lambda _{\text {endo}}$ and *p* by the same factor. In order to recover the correct absolute values of pressures and parameters, we can use a single known cavity pressure at a specific time point in the cardiac cycle. An example would be the peak systolic LV pressure (SP) estimated from cuff measurements using a Centron cBP301 device, which transforms acquired peripheral pressures to compute central pressures (Brett et al. 2012). In systole, when the aortic valve is open, the central pressure estimate can be used as an accurate LV pressure estimate. Alternatively, an end-diastolic pressure (EDP) estimate can be obtained from the $E/E_a$ ratio (Nagueh et al. 1997, 2009). *E* is the peak early-diastolic flow velocity through the mitral plane and can be measured in phase contrast MR sequences. $E_a$ (sometimes denoted by $E'$, or $e'$) is the early-diastolic velocity of the mitral annulus on the lateral side of the base and can be computed using the tracked motion of the mesh.

Moreover, both the EDP and the SP estimates, along with the timings of mitral and aortic valve opening and closing, can be used to generate a full-cycle patient-specific LV pressure curve from a normalised trace obtained in Russell et al. (2012), shown to be consistent across a range of cardiac disorders. Even though the accuracy of such pressure data, and hence its use as a driving force in the model, is limited (compared to the accuracy of image-derived cavity volumes), matching pressures along with displacements in the active estimation process proves beneficial (see Sect. 4).

The types of clinical data used in the simulation and estimation process in the in vivo tests in Sect. 3.2 were:

1. short-axis cine to produce the computational mesh,
2. 3D tags to extract mesh deformations,
3. pressure cuff measurement to estimate the SP,
4. mitral flow velocity to estimate the EDP.

The datasets were acquired as part of the BHF New Horizons project *Integrated Mathematical Modelling and Imaging for Dilated Cardiomyopathy* (DCM). Specifically, we processed one dataset from a healthy volunteer and two datasets from patients being treated for moderate DCM-related heart failure. The cine, 3D tagged and flow images were acquired on a 1.5T Philips Achieva system with the following specifications:

- cine bSSFP in retrospective ECG gating, spatial resolution $2\times 2\times 8$ mm, temporal resolution $\sim$20 ms, FOV $350\times 350$ mm.
- 3D tagged MRI in prospective ECG triggering, spatial resolution $3.4\times 7.7\times 7.7$ mm, temporal resolution $\sim$30 ms, FOV $100\times 100\times 100$ mm, reconstructed interpolated image with spatial resolution $1\times 1\times 1$ mm.
- 4D flow^2^ in prospective ECG triggering using an MRI breathing navigator, spatial resolution $2.3\times 2.3\times 2.3$ mm, temporal resolution $\sim$35 ms, velocity encoding range 100–150 cm/s.

**Fig. 1:**
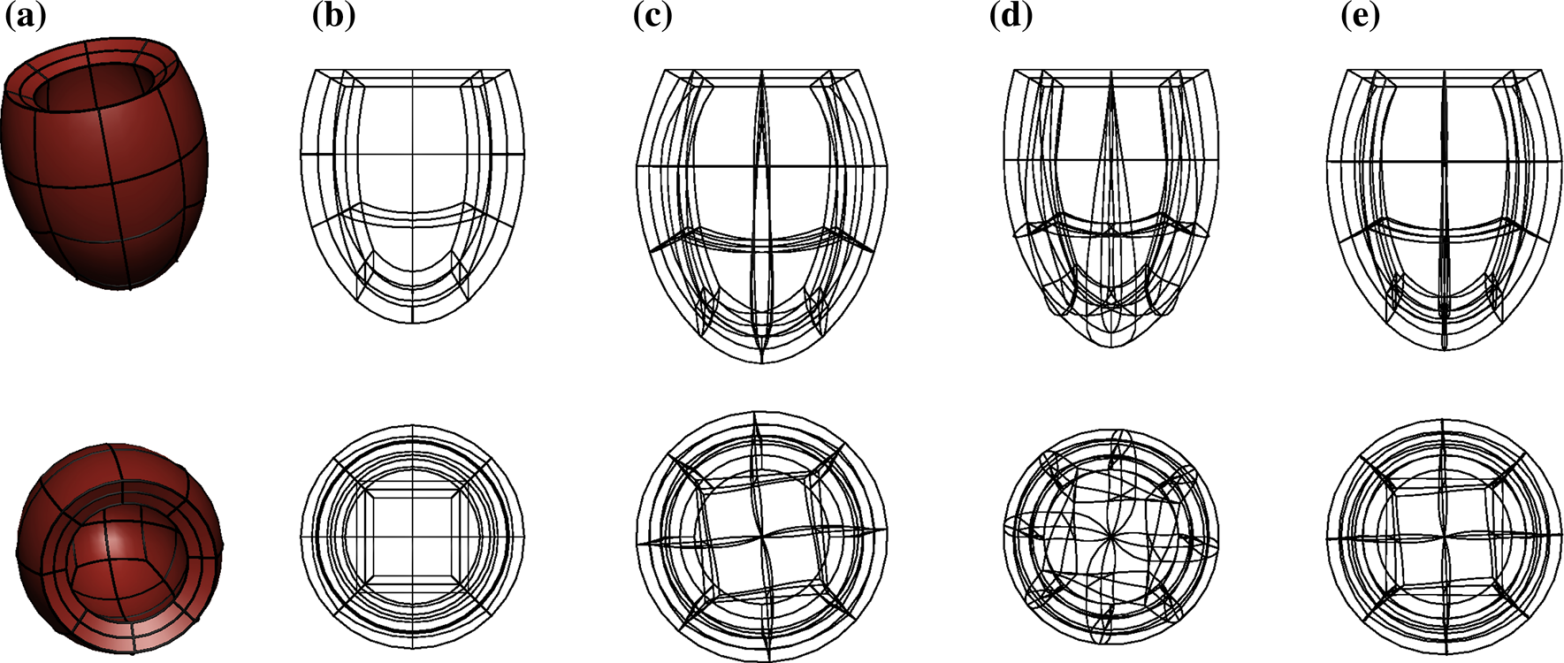
Idealised geometry in undeformed and deformed states through the cardiac cycle. **a** Reference geometry, **b** reference mesh, **c** end-diastolic mesh, **d** mid-systolic mesh, **e** end-systolic mesh

An end-diastolic LV mesh was created using the CGAL library^3^ (The CGAL Project 2015) from a CardioViz3D^4^ (Toussaint et al. 2008) segmentation of the end-diastolic frame of the short-axis cine image. The mesh was deformed using the results of IRTK motion tracking (Shi et al. 2013) from the 3D tagged image set covering the majority of the cardiac cycle. The mesh with the lowest LV cavity volume was used as the reference state for the simulations. The distances between the deformed meshes and this reference mesh at all time steps served as reference displacement data.

The end-diastolic pressure was estimated using the $E/E_a$ ratio: $\lambda _0=1.9+1.24 E/E_a$ (Nagueh et al. 1997), with the peak early-diastolic flow velocity through the mitral plane *E* estimated using GyroTools GTFlow software^5^ from 4D flow images, and the early-diastolic velocity $E_a$ computed at the lateral side of the base from reference displacements (assumed to be sufficiently close to the mitral annulus). The peak systolic pressure was estimated from cuff measurements using a Centron cBP301 device^6^ (Brett et al. 2012). The full-cycle pressure curve was obtained by scaling the normalised pressure curve between $\lambda _{\max }$ at maximum and $\lambda _0$ at the time corresponding to the first frame of the 3D tagged sequence. The time scaling was based on valve events extracted from the mesh volume curve, so as to obtain a physiological shape of the reference P–V loop.

Building the personalised models for the described in vivo datasets allows us to establish the potential applicability of the pipeline to clinical data and the feasibility of interpreting the estimated material parameters as characteristic of tissue properties, and consequently cardiac health and disease.

### In silico datasets

The evaluation of the proposed pipeline using typical clinical datasets is the ultimate test of its applicability. However, such evaluation is often obscured by variable data quality. The lack of ground truth for most computed variables as well as model parameters means that validation using medical images is somewhat limited in scope. In silico testing, whereby the datasets are generated using the model, provides a useful tool for bridging the gap between model and clinical measurement. In this case, when all of the reference parameters are known and the model has perfect fidelity, we can investigate the accuracy of the estimation procedure as well as its sensitivity, specifically the effects of noise in the data and motion tracking errors on the quality of parameter estimates.

An idealised left ventricle was modelled as an ellipsoid cropped at an angle at the base, as shown in Fig. 1a. This geometry can be easily generated for testing purposes and provides a straightforward point for comparison. The idealised fibre field was produced as described for the in vivo datasets.

**Fig. 2:**
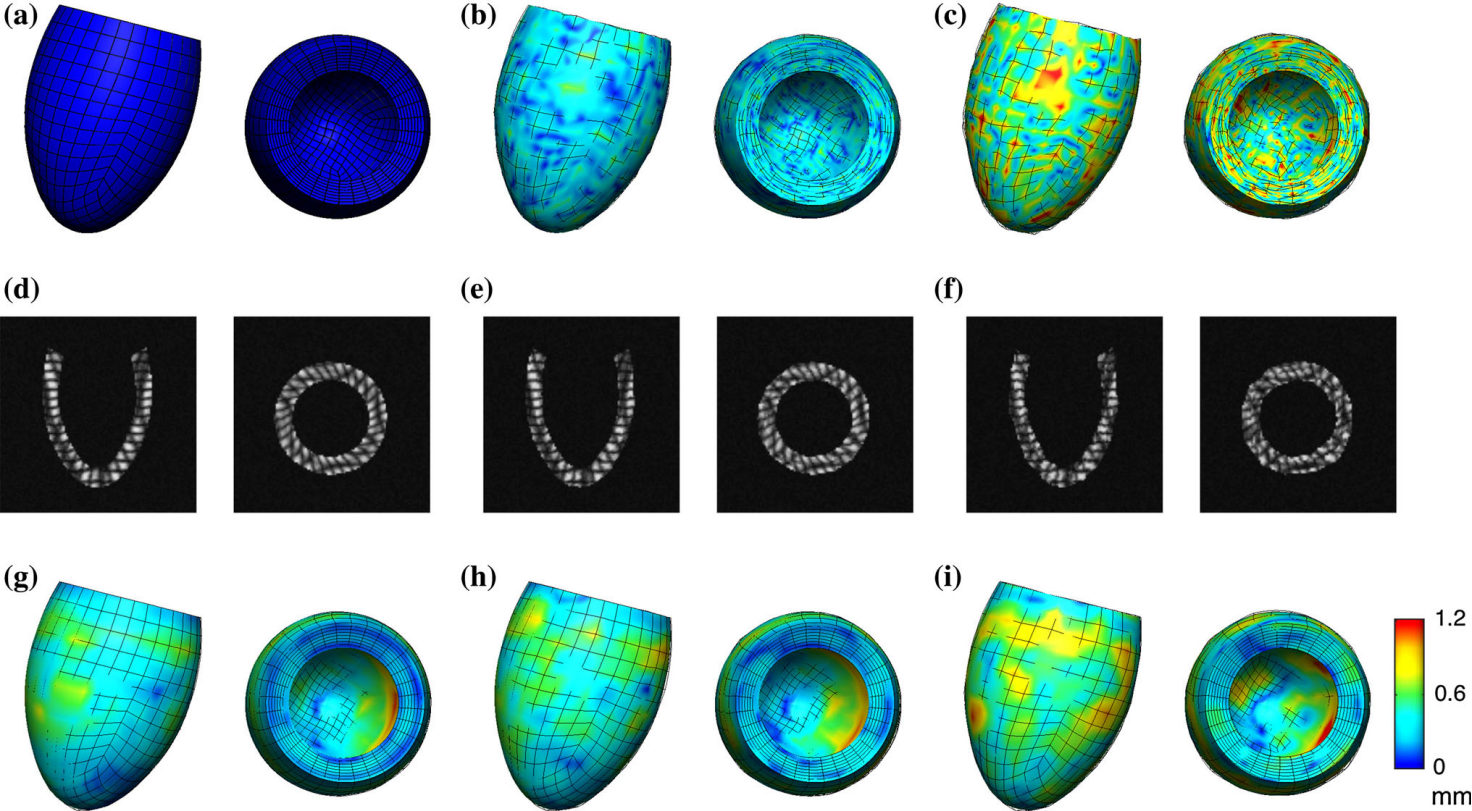
Comparison of in silico datasets in mid-systole (long- and short-axis views). The surfaces show the domain deformed using clean displacement data, while the lines crossing the surfaces show the mesh deformed using the six different datasets produced for testing: clean unprocessed, unprocessed with noise at 10 and 20 % standard deviation, clean processed and processed noisy at 10 and 20 % standard deviation. Surface shading represents the distance between the clean displacements and the displacement for each of the cases. **a** Clean, **b** noisy, 10 % std, **c** noisy, 20 % std, **d** clean tags, **e** noisy tags, 10 % std, **f** noisy tags, 20 % std, **g** processed clean, **h** processed noisy, 10 % std, **i** processed noisy, 20 % std

The displacement data were produced in three stages, as illustrated in Fig. 2. First, we obtained *clean displacements* (Fig. 2a) by running a full-cycle simulation using a given volume curve with given reference parameters $a^{\text {ref}}, a_f^{\text {ref}}$ and a given active scaling function $\alpha ^{\text {ref}}(t)$. The input data were chosen so that the model produced physiological cavity pressures, P–V loop and LV deformations. The mesh underwent characteristic twist and shortening (Fig. 1d) in systole before relaxing (Fig. 1e) and inflating (Fig. 1c) in diastole.

Second, random zero-mean uniform noise with a given standard deviation was added to clean displacements to produce *noisy displacements* (Fig. 2b, c). The random nature of the added noise means that its spatial distribution is not in any way related to any local geometric features of the mesh. This ensures that the symmetry of the idealised geometry does not artificially improve parameter identifiability or the accuracy of estimation. Third, we generated artificial 3D tagged images (Fig. 2d–f) using clean and noisy displacements and performed motion tracking as described above to extract *processed displacements* from the images (Fig. 2g–i).

The full pressure curve was produced as simulation output together with clean displacements.

### Model personalisation process

The full-cycle estimation process is carried out via the following steps:

1. estimation of the passive parameter ratio from diastolic displacements,
2. scaling the passive parameters and estimated pressures using the reference EDP,
3. estimation of the active tension from a combination of the displacements and LV cavity pressures through the cycle.

The procedure is aimed at finding parameters that produce simulation results closest to the data, as measured by *objective functions*${\mathcal {J}}$.

Let ${\varvec{u}}_n^{\text {ref}}$ and $\lambda _n^{\text {ref}}, n=0,\ldots ,N$, be the reference displacements and cavity pressures, respectively, $n=0$ corresponding to end diastole and $n_m$ to end systole.

Since the passive parameters *a* and $a_f$ are assumed to be constant in time, the objective function in the passive stage is defined as the relative total displacement error:

9 $$\begin{aligned} {\mathcal {J}}_p[{\varvec{u}}] = \left( \frac{\displaystyle \sum _{n = m+1}^N \Vert {\varvec{u}}_n - {\varvec{u}}_n^{\text {ref}}\Vert ^2 }{\displaystyle \sum _{n = m+1}^N \Vert {\varvec{u}}_n^{\text {ref}} \Vert ^2}\right) ^{1/2}, \end{aligned}$$

with $\Vert \cdot \Vert$ the standard $L^2(\varOmega _0)$-norm. The effect of residual active tension at the start of diastole is neglected due to our inability to reliably deduce its presence in vivo. This assumption can be corrected for based on active estimation: if the active scaling is estimated at a significant positive value in the initial diastolic frames, then the passive parameters can be adjusted based on estimation in the remaining diastolic frames (see Sect. 4).

As discussed above, in volume-driven simulations the pressure solutions scale together with *a* and $a_f$, so that displacements alone do not identify both passive parameters, only the ratio between them $\gamma = a/a_f$. In practice, a fixed value of $a_f^{\text {sim}}$ can be used to find $a^{\text {sim}}$ which minimises the objective function.^7^ The correct absolute values of parameters *a* and $a_f$, as well as estimated cavity and myocardial pressures $\lambda _{\text {endo}}$ and *p*, are recovered by scaling the respective values used or computed in the simulations by the ratio of the reference to the estimated EDP $\lambda _0^{\text {ref}}/\lambda _0^{\text {sim}}$. In the current procedure, this scaling will also be consistent with the peak systolic pressure, since the personalised full-cycle pressure curve is scaled to both the EDP and the SP. The correct absolute values of the base pressure ${\varvec{\lambda }}_{\text {base}}$ can be obtained by running inflation with the correct absolute parameter values.

**Fig. 3:**
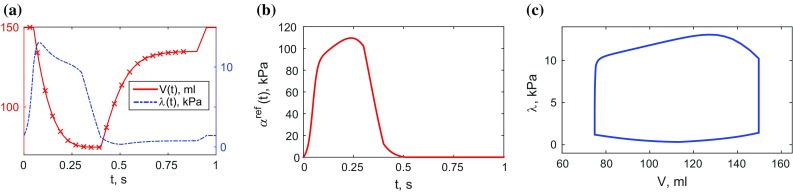
Reference data used in in silico tests: volume and active tension curves are constructed based on typical behaviour, and pressure curve is computed using the model. The *crosses* on the volume curve correspond to 3D tagged image frames. **a** Volume and pressure, **b** active tension, **c** P–V loop

In the active stage, the parameter $\alpha$ is estimated through time, or, more precisely, at times when displacement data are available: $\alpha _n, n=1,\ldots ,N$. The objective function is computed per time step and incorporates both relative displacement and pressure errors:

10 $$\begin{aligned} {\mathcal {J}}_a[{\varvec{u}}_n,\lambda _n] = 0.5\frac{\Vert {\varvec{u}}_n-{\varvec{u}}_n^{\text {ref}}\Vert }{ \displaystyle \max _{n=0,\ldots ,N}\Vert {\varvec{u}}_n^{\text {ref}}\Vert } + 0.5\frac{|\lambda _n-\lambda _n^{\text {ref}}|}{|\lambda _n^{\text {ref}}|}, \end{aligned}$$

for $n=0,\ldots ,N$. Equal weighting is given to relative displacement and pressure errors in the combined functional in order to balance the uncertainty in the two variables, but this can be adjusted if deemed necessary. The displacement errors are taken in relation to the maximum over all frames, not current displacement, in order to neutralise the effect of zero and low displacements at and around end systole.

In addition to computing objective functions which measure relative errors in problem variables, for the in silico tests, we can compute the relative errors in the parameter estimates:

11 $$\begin{aligned} e_p[\gamma ]&= \frac{|\gamma - \gamma ^{\text {ref}}|}{\gamma },\end{aligned}$$

12 $$\begin{aligned} e_a[\alpha _n]&= {\left\{ \begin{array}{ll} \displaystyle \frac{|\alpha _n - \alpha _n^{\text {ref}}|}{\alpha _n^{\text {ref}}}, &{} \alpha _n^{\text {ref}} \ne 0, \\ \max (\alpha _n,0), &{} \alpha _n^{\text {ref}}=0. \end{array}\right. } \end{aligned}$$

These metrics are useful in assessing the quality of the estimation and the sensitivity of the model.

## Results

First, we describe test specifications (including geometry, input data, parameters and generation of clean reference data) and present results for the in silico cases. Model outputs were compared to reference data, and the effects of noise and tags processing were assessed. Next, we describe the in vivo datasets processed and present pipeline outputs for these cases. All simulations were performed using $\varvec{\mathcal {C}}$**Heart** (McCormick et al. 2011, 2013),^8^ a multiphysics solver package developed at the Biomedical Engineering Department, King’s College London.

**Fig. 4:**
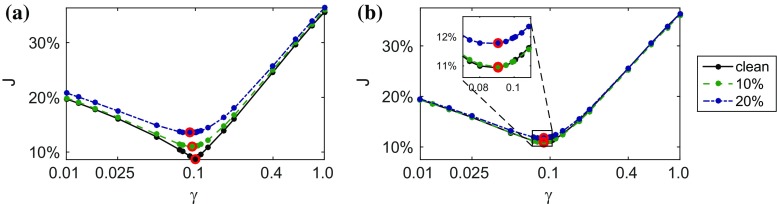
Variation of the objective function ${\mathcal {J}}_p$ over the space of the passive parameter ratio $\gamma (\gamma ^{\text {ref}}=0.1)$. The position of the minimum is indicated by a *circle*. Each data point corresponds to a single simulation run. **a** Unprocessed, **b** processed

### In silico cases

The computational mesh was composed of 56 quadratic hexahedral elements.^9^

The cycle was taken to last 1000 ms, driven by the cavity volume curve shown in Fig. 3a and the active tension curve shown in Fig. 3b. The reference parameters were chosen to be $a^{\text {ref}}=0.16$ kPa and $a^{\text {ref}}_f=1.6$ kPa, with $\gamma ^{\text {ref}} = 0.1$. For simplicity, the base of the ventricle was assumed to be fixed. The cavity pressures produced by the reference simulation are shown in Fig. 3a, and the reference P–V loop in Fig. 3c.

Reference simulation results were obtained at 5-ms intervals to achieve convergence of the nonlinear solver. However, only solutions at $30, 70, \ldots , 830$ ms were taken as data points, making a total of 21 “frames” to emulate a 3D tagged image sequence typically obtained using the in vivo imaging protocol by means of temporal sampling and initial offset given by the prospective ECG triggering.

Two sets of unbiased uniform noise were generated for each set of clean data: with standard deviations of 10 and 20 % of maximum displacement length, where the maximum was taken for each displacement to end-diastolic state (ranging between 6 and 12 mm).

#### Estimation of the passive parameters

All diastolic frames ($t= 510, 550, \ldots , 830$ ms) were used for the estimation of the passive parameter ratio. In addition, the first frame ($t_0= 30$ ms) was assumed to be sufficiently close to the end-diastolic state to provide a data point at $t=1000$ ms. Since the reference volume changes between frames were too large to apply in single simulation steps, additional linearly interpolated volumes were used. Even though the exact reference volumes were known at intermediate steps between frames, they were not used in estimation, so that the dataset resembled the in vivo case.

For each of the six datasets, a parameter sweep consisting of $\sim$25 simulations was carried out in order to assess the behaviour of the objective function ${\mathcal {J}}_p$ over the $\gamma =a/a_f$ space (see Fig. 4) and find its minimum (expected at or near $\gamma ^{\text {ref}}=0.1)$. The fibre parameter was fixed at $a_f^{\text {sim}}=1$ kPa in all simulations.

The estimated values of $e_p[\gamma ]$ are given in Table 1 with uncertainty ranges determined by sweep intervals.

**Table 1:** Relative errors in parameter estimates $e_p[\gamma ]$ with ranges determined by parameter sweep intervals

Level of noise	0 % (clean)	10 %	20 %
Unprocessed	$(0\pm 1)$ %	$(7\pm 1)\%$	$(11\pm 1)\%$
Processed	$(13\pm 1)$ %	$(12 \pm 1)\%$	$(14\pm 1)\%$

Scaling the estimated $a^{\text {sim}}, a_f^{\text {sim}}, \lambda _{\text {endo}}^{\text {sim}}$ and $p^{\text {sim}}$ by $\lambda _0^{\text {ref}}/\lambda _0^{\text {sim}}=1.6$ allowed us to recover the exact EDP.

#### Estimation of the active tension

**Fig. 5:**
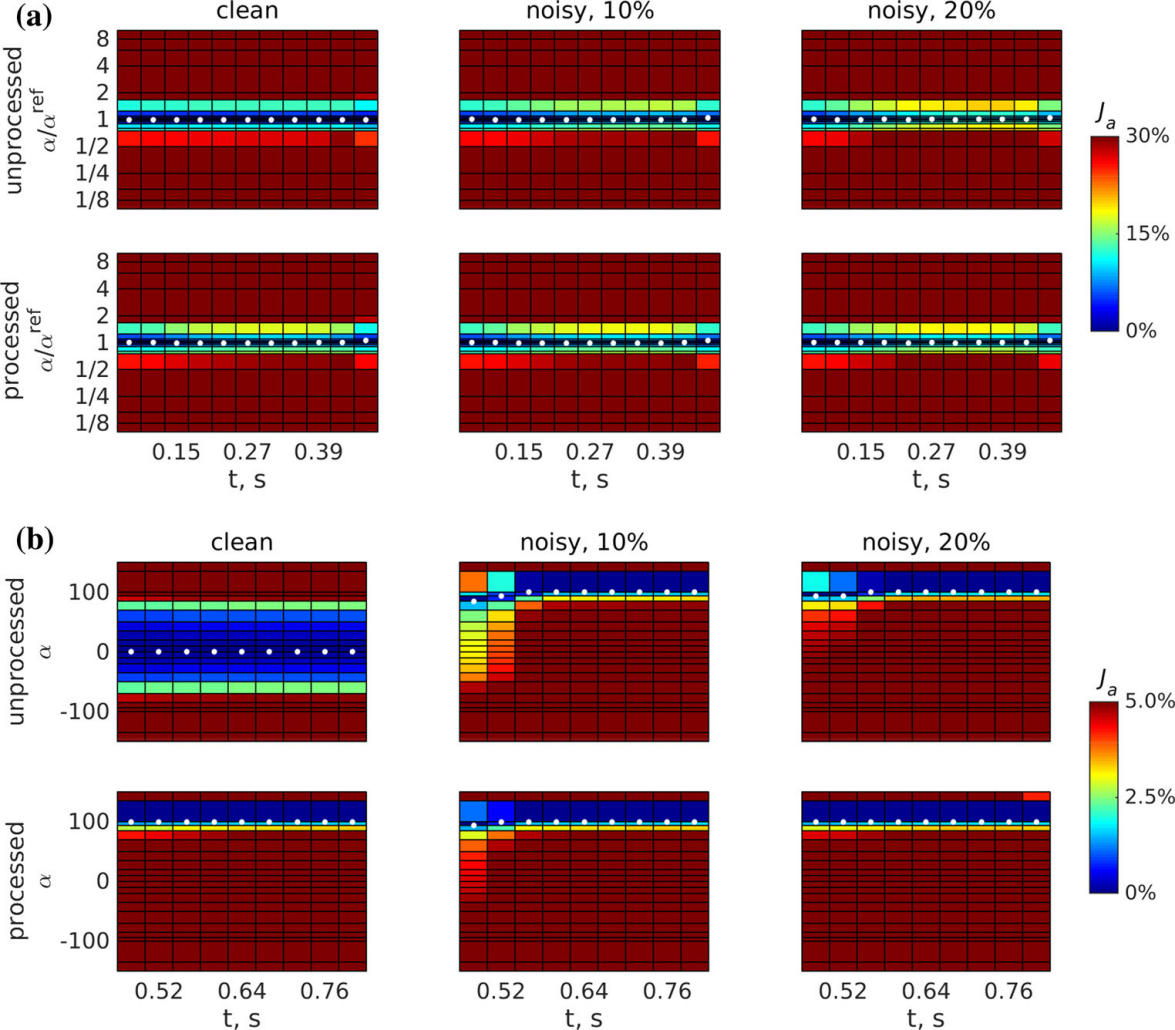
Variation of the objective function ${\mathcal {J}}_a$: **a** over the $\alpha /\alpha ^{\text {ref}}$ space in systole, when $\alpha ^{\text {ref}}>0$, and **b** over the $\alpha$ space in diastole, when $\alpha ^{\text {ref}}=0$. *White dots* indicate positions of the minima for each step

**Fig. 6:**
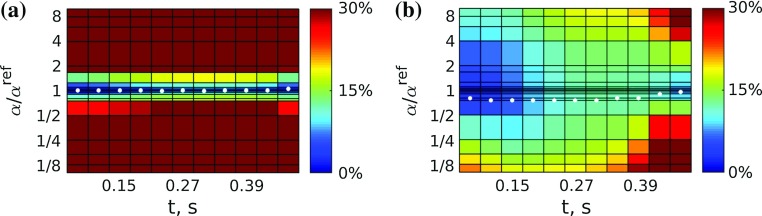
Comparison of the variation of **a** the objective function ${\mathcal {J}}_a$ combining relative displacement and pressure errors, and **b** the objective function consisting of relative displacement errors only for processed displacements with 20 % noise. *White dots* indicate positions of the minima for each step. Sharper variation around the minimum translates to better identifiability of the active parameter $\alpha$. **a** Displacement and pressure, **b** displacement only

The estimated passive parameters for each dataset were used in active scaling estimation. We performed parameter sweeps for each time frame in order to map the behaviour of the active objective function. In the systolic frames with nonzero reference active tension $\alpha _n^{\text {ref}}>0$ at $t_n=70, 110, \ldots , 470$ ms, parameter sweeps were carried out around the reference values $\alpha _n^{\text {ref}}$. For each time step, cavity volume was fixed at the respective reference value, and active tension was increased from $0.1\alpha ^{\text {ref}}$ to $10\alpha ^{\text {ref}}$ over 21 steps. The variation of the objective function at each time step over this range of $\alpha$ is shown in the $\alpha /\alpha ^{\text {ref}}$ space in Fig. 5a. The minimum was always expected at or near $\alpha /\alpha ^{\text {ref}} = 1$. A sample plot for processed data with 20 % noise is shown in Fig. 6 comparing the behaviour of the objective function ${\mathcal {J}}_a$ with a displacement-only objective $\Vert {\varvec{u}}_n-{\varvec{u}}_n^{\text {ref}}\Vert /\Vert \max _n{\varvec{u}}_n^{\text {ref}}\Vert$.

For diastolic frames with zero reference active tension $\alpha _n^{\text {ref}}=0$ at $t_n=510, 550, \ldots , 830$ ms, parameter sweeps consisting of 21 simulations were carried out between $-$1 and 1 kPa, and the variation of the objective function is shown in the $\alpha$ space in Fig. 5b. In this case, the minimum was always expected at or near $\alpha = 0$.

The mean and maximum errors in the active scaling estimated using the combined objective function $J_a$ are given in Table 2 for the systole and Table 3 for the diastole.

**Fig. 7:**
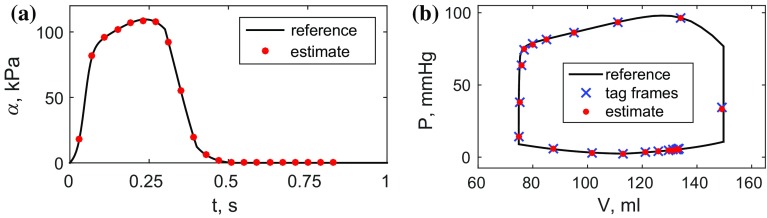
Comparison of the clean reference data and estimates for **a** the active tension scaling and **b** the P–V loop for the processed displacements with 20 % noise. **a** Active scaling, **b** P–V loop

Sample comparison plots (worst case, processed displacements with 20 % noise) for the reference and estimated active scalings and P–V loops are shown in Fig. 7a, b.

### In vivo cases

We processed three datasets acquired in a healthy volunteer (**V**) and two patients with dilated cardiomyopathy (**P1** and **P2**). Case details are presented in Table 4.

**Fig. 8:**
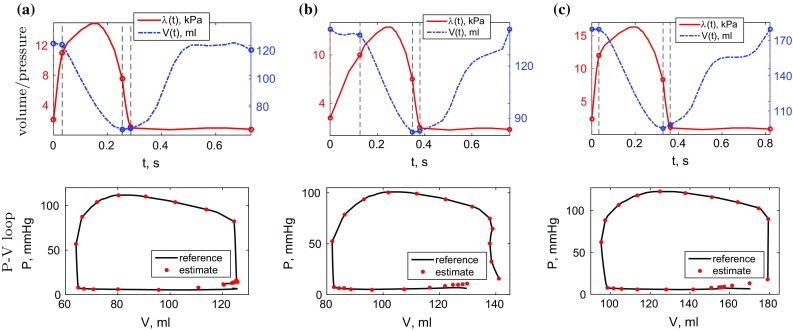
Reference volume data for the in vivo cases computed from the deformed meshes, and reference pressure data obtained by scaling the normalised curve (Russell et al. 2012) using mitral and aortic valve opening and closing times (*vertical dashed lines*). *Dots* on the P–V loop figures indicate the values produced by the model

The meshes were composed of 7795 (**V**), 10740 (**P1**) and 17047 (**P2**) linear tetrahedral elements. The reference volumes were computed from meshes deformed according to 3D tagged image tracking, and reference pressure curves were scaled between estimated end-diastolic and peak systolic pressures, and in time based on volume-derived valve events in order to obtain physiological shapes of the reference P–V loops (see Fig. 8).

Weak adherence to reference base displacements was required in all simulations ($\varepsilon =10^{-7}{-}10^{-8}$), and the reference mesh volumes were used in the endocardial boundary condition.

**Table 2:** Relative errors in parameter estimates $e_a[\alpha _n]$ (**mean**/max) for systole when $\alpha _n^{\text {ref}}>0$

Level of noise	0 % (clean)	10 %	20 %
Unprocessed	$\varvec{0}/0$ %	$\varvec{1}/5\%$	$\varvec{1}/5\%$
Processed	$\varvec{0}/5$ %	$\varvec{0}/5\%$	$\varvec{0}/5\%$

**Table 3:** Absolute errors in parameter estimates $e_a[\alpha _n]$ (**mean**/max) for diastole when $\alpha _n^{\text {ref}}=0$

Level of noise	0 % (kPa)	10 % (kPa)	20 % (kPa)
Unprocessed	$\varvec{0}/0$	$\varvec{0.9}/1$	$\varvec{0.94}/1$
Processed	$\varvec{0.1}/0.1$	$\varvec{0.92}/1$	$\varvec{1}/1$

Estimated active tension does not exceed 1 kPa, which is less than 1 % of the peak active tension, allowing a differentiation between the passive and the active stages of the cycle

**Table 4:** Basic information for the datasets used in the in vivo tests

	**V**	**P1**	**P2**
Case descriptions			
Gender	M	F	M
Age	41	28	55
HR (bpm)	64	58	63
Data-derived metrics			
EDV (ml)	120	141	179
ESV (ml)	54	82	95
EF (%)	56	42	47
SP ($\lambda _{\max }$) (mmHg)	112	101	122
*E* (cm/s)	97	107	103
$E_a$ (cm/s)	13	9	8
EDP ($\lambda _0$) (mmHg)	11.3	16.3	17.6

*HR* heart rate, *EDV/ESV* end-diastolic/end-systolic volume, *EF* ejection fraction, *SP* peak systolic pressure, *EDP* end-diastolic pressure

#### Estimation of the passive parameters

**Fig. 9:**
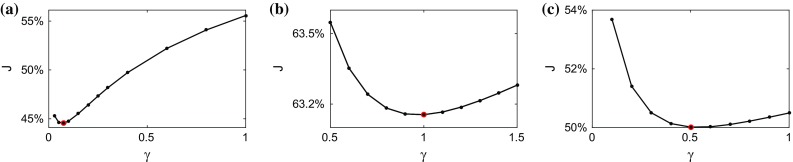
Variation of the objective function ${\mathcal {J}}_p$ over the passive parameter ratio space in the in vivo cases, with minima shown as *circles*

As before, estimation of the passive parameter ratio was carried out as a parameter sweep, refining the sweep intervals in the area of lower ${\mathcal {J}}_p$. The fibre parameter was fixed at $a_f=1$ kPa. All diastolic frames were used in computing the objective function, as well as the end-diastolic state obtained from the first frame of the 3D tagged images. Figure 9 shows the variation of ${\mathcal {J}}_p$ over a range of passive parameter ratios $\gamma$.

The minimising ratios $\gamma$ along with the passive parameter values *a* and $a_f$ scaled by the ratios of the reference and the simulated EDPs are shown in Table 5.

**Table 5:** Estimation results for the in vivo cases. $\alpha _{\max }$ denotes the peak active tension, ${\hat{t}}_{\max }$ the scaled time to peak activation: ${\hat{t}}_{\max } = t_{\max } \cdot (\text {HR}) / 60$, and $\min _t$ and $\max _t {\mathcal {J}}_a$ refer to the lowest and highest absolute values of the objective function across all systolic frames at the minimum over $\alpha$

	**V**	**P1**	**P2**
Passive estimation			
$\gamma$	0.07	1.00	0.50
*a* (kPa)	0.274	2.186	1.291
$a_f$ (kPa)	3.909	2.186	2.582
Active estimation			
$\alpha _{\max }$ (kPa)	139	145	191
${\hat{t}}_{\max }$ (ms)	0.23	0.28	0.24
$\min _t {\mathcal {J}}_a$ (%)	24	15	13
$\max _t {\mathcal {J}}_a$ (%)	35	28	24

#### Estimation of the active tension scaling

**Fig. 10:**
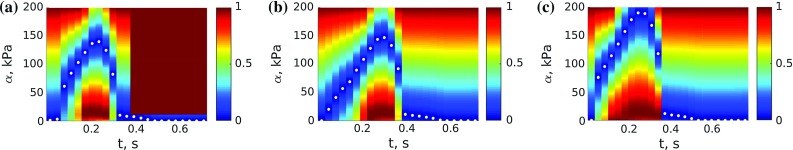
Variation of the objective function ${\mathcal {J}}_a$ over the $\alpha$ space for each time step in the in vivo cases, with minima shown as *white dots*. Each *vertical line* corresponds to a single ${\mathcal {J}}_a$ over $\alpha$ plot for a given time *t*. For clear presentation of the minima in a combined plot over all time steps, the values of ${\mathcal {J}}_a$ were scaled to [0,1] for each *t*, and the shading corresponds to these scaled values. The range of the absolute values of ${\mathcal {J}}_a$ is given in Table 5. **a** **V**, **b** **P1**, **c** **P2**

A parameter sweep was carried out at each time step corresponding to a frame of the 3D tagged image. At a given time step, a simulation was carried out whereby the active tension scaling increased from 0 to 200 kPa using 1 kPa increments (i.e. each simulation took 200 parameter increment steps). The variation of ${\mathcal {J}}_a$ over the parameter space for each time step is shown in Fig. 10. The lowest and highest absolute values of the combined displacement and pressure objective function in systole together with peak estimated active tension and the relative time to peak activation are given in Table 5 for all cases.

**Fig. 11:**
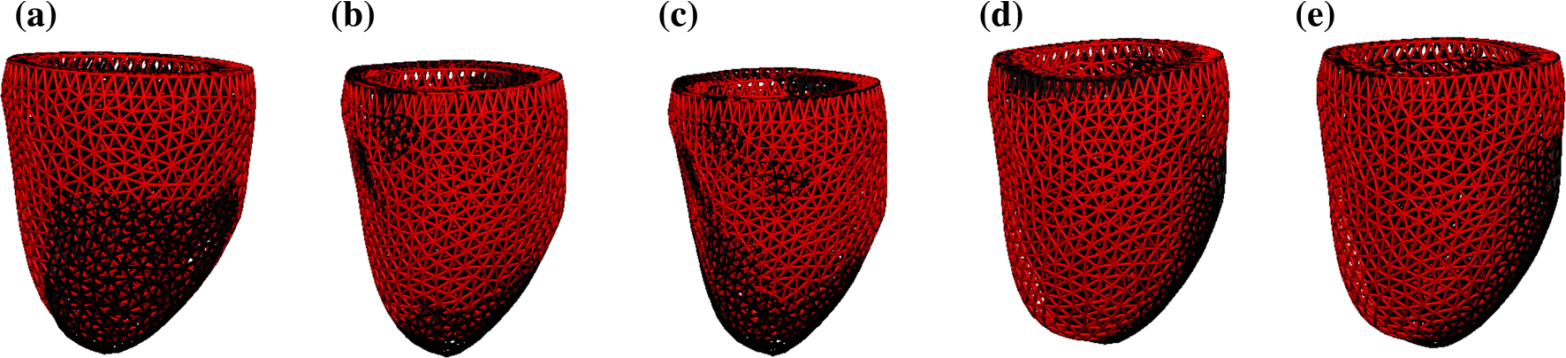
Mesh configurations through the cycle based on the model with estimated passive and active parameters (*black*) and motion tracking in the 3D tagged image set for case **V**. **a** End diastole, **b** mid-systole, **c** end systole, **d** mid-diastole, **e** late diastole

Figure 11 shows the reference mesh configurations for case **V1** at different stages of the cardiac cycle based on 3D tagged motion tracking alongside simulation results obtained using estimated parameters.

## Discussion

Patient-specific cardiac modelling holds significant promise for providing details on the performance, function and properties of the heart. However, the process of personalising mechanical models needs to be reliable and robust in order to become a viable tool for model-based analysis of patient cases. The modelling pipeline proposed in this work is based entirely on a non-invasive dataset, which includes a short-axis cine MRI stack (used to construct the LV mesh) and a 3D tagged MRI sequence (providing mesh deformations over time) as well as end-diastolic and end-systolic pressure estimates derived from flow MRI and a cuff pressure measurement. The main goal of the study was to ensure the existence of unique sets of parameters that provide a good fit to the three-dimensional cardiac motion over the cardiac cycle. The presented model aims to balance model fidelity with parameter identifiability, producing mechanical equations that depend linearly on the parameters. Identifiability was verified and validated by sweeping parameter spaces in a series of tests where in silico and in vivo data were matched to model predictions. Unique parametrisation was possible in all cases, providing two constant stiffnesses (defining passive behaviour) and a time-dependent active tension (driving the active contraction) estimated based on a rich set of displacements across the myocardium and an LV cavity pressure curve estimate ensuring physiological behaviour of the model. While the constitutive laws used were highly nonlinear with respect to problem variables, their dependence on the estimated parameters was linear, resulting in each of the objective functions ${\mathcal {J}}$ having a single global minimum. In all cases, model fidelity was sufficient to retain this property of ${\mathcal {J}}$ ensuring our ability to estimate reliable parameters.

### In silico cases

Testing with in silico datasets provided a useful insight into how the proposed model and parameterisation performed. The in silico tests (see Fig. 2) enabled a decoupling between the issues of model fidelity and parameter identifiability, since the model was tested under the circumstances where it was able to replicate reference deformations. The knowledge of the reference parameters used for generating the data allowed the assessment of the sensitivity of the model parameters to different types of errors (in addition to the sensitivity of the problem variables).

In active estimation, the identification of active tension scaling throughout the cardiac cycle was uniformly achieved (see Fig. 5). The inclusion of random displacement noise of 10–20 % resulted in a minimal increase in error, while errors in pressure values translated directly to active parameter errors. Activation through diastole was also minimal, showing a bias towards activation using processed data (though this was below 0.01 % peak tension). It is interesting to note that identification of myocardial activation was achievable from displacement data alone (see Fig. 6), suggesting that, given sufficient model fidelity, a full-cycle pressure curve would not be required for estimation. However, the objective function based on both displacement and pressure errors provided improved accuracy (max 5 %, average 0.5 % error for 20 % noise in data) compared to that based on the displacement term alone (max 25 %, average 20 % error for 20 % noise in data). In the latter case (which would allow full-cycle estimation based on a single pressure measurement), peak systolic pressure was underestimated by 25 %. While scaling using the measured to estimated pressure ratio would adjust the peak active tension to the correct value, it would also overestimate both passive parameters by an additional $\sim$33 %, as well as overestimate the active tension curve away from the peak up to $\sim$33 %.

### In vivo cases

Following the pipeline introduced in Sect. 2.2, a unique parametrisation of the model was obtained for each of the volunteer and patient datasets acquired non-invasively. The myocardial geometry was obtained from the short-axis cine stacks registered to 3D tagged images and tracked through the cycle using IRTK, providing observations for estimation as well as boundary condition data, namely LV cavity volumes (see Fig. 8) and basal deformations. Following the parameterisation process, Figs. 9 and 10 show that unique identification of the passive ratio $\gamma$ and active tension scaling $\alpha (t)$ was achieved. Moreover, Fig 11 illustrates that qualitative model motion corresponded well with extracted motion from the patient data.

The active estimation results indicated that residual active tension was present in the myocardium in 4–5 frames past end systole. This additional information could be used to correct passive parameter estimates. For example, the objective function ${\mathcal {J}}_p$ was recomputed for case $\mathbf{V}$ based on the frames with no residual tension (15–24), and the minimum was achieved at $\gamma =0.08$. After scaling by end-diastolic pressure ratio, the corrected parameter values were $a=0.304$ kPa and $a_f=3.8$ kPa, including relative corrections of 10 % for *a* and 3 % for $a_f$.

Unlike the in silico tests, where good model fidelity allows estimation of the active tension based solely on the tracked myocardial data, the in vivo cases relied on the pressure curve in producing a viable active tension estimate. The minimum of the displacement component of the active functional was achieved at near-zero active tension throughout the cardiac cycle for all datasets due to model errors dominating the term. The use of non-invasive pressure estimates ensured that a reasonable pressure–volume loop was produced by the model (see Fig. 8).

While we cannot make reliable interpretation of parameters based on three datasets only, some differences can already be observed in the results in Sect. 3.2. The estimated passive parameter ratio is significantly lower for the healthy volunteer, and consequently we estimate that the tissue is much stiffer in the fibre direction than isotropically, which does not appear to be the case for the DCM patients. Case **P1** activates significantly slower, and case **P2** significantly higher than **V**. The higher total relative error ${\mathcal {J}}_p$ and the shallower curves near the minimum in the passive estimation for $\mathbf{P1}$ and $\mathbf{P2}$ as compared to $\mathbf{V}$ could be caused by reduced motion of the diseased myocardium and therefore a stronger effect of model errors. Identifiability is not affected in the active estimation due to reliance on the same normalised pressure curve. A detailed analysis of estimation results for a wider range of healthy and diseased cases would be fundamental for building an understanding of the link between the estimated quantities and physiology.

### Limitations and future work

In the current study, a range of in silico datasets and three in vivo cases are considered in order to validate unique identifiability of parameters in the personalisation process. A comparative analysis of parameter estimates for a wider range of cases including healthy volunteers and patients is necessary for determining the diagnostic and prognostic potential of the model parameters as biomarkers. While personalisation of multiple in vivo datasets using parameter sweeps would incur a high computational cost, the results of this work suggest that commonly used optimisation methods, such as the reduced-order unscented Kalman filter (Chabiniok et al. 2012; Moireau et al. 2008; Xi et al. 2011), would perform well in the same parameter spaces using the outlined objective functions.

A direct comparison of the in vivo parameter estimates with results available in the literature for the Holzapfel–Ogden law would be problematic due to differences in some of the basic assumptions on fixed parameter values taken in different studies. Further effort in this direction can be made based on recent experimental results in Sommer et al. (2015).

The objective functions used to determine the optimal parameters for a given case took advantage of the local information on the myocardial position over the cardiac cycle extracted from 3D tagged images. To our knowledge, this is the first instance when the relative $L_2(\varOmega _0)$-norm of the error between myocardial deformations obtained using image processing and model predictions has been assessed and reported. In the passive parameter estimation, the error is accumulated over time as well as space, leading to the values of the objective function $\min {\mathcal {J}}_p = 45$–63 %. Active estimation relies on a combined displacement and pressure error functional, with the pressure component ensuring parameter identifiability throughout the cycle.

The strictness of the objective function accumulating all local errors can partly explain the relatively high values of ${\mathcal {J}}$. Nevertheless, further adjustment of the model should be sought to improve fidelity and specifically to reduce displacement errors. In particular, local errors were largest in the septum and at the right-ventricular attachment points, suggesting that the influence of the right ventricle should be accounted for when considering left-ventricular mechanics. Additional tests have been carried out to assess the potential for reducing model errors (thus improving model fidelity) by using different fibre distributions in the myocardium, or introducing a transverse activation law (see “Appendix 2”). As may be expected, the absolute values of the parameters obtained using these modified models change to some extent. More importantly, the identifiability of parameters is not dramatically affected by either modification. The viability of using regional passive parameters and non-homogeneous activation should be investigated.

The choice of the end-systolic geometry as the reference state is likely to have an effect on displacement errors, as well as passive estimation results. An investigation of the bias introduced by this choice would be of interest. While the present set-up did not permit simultaneous estimation of the passive parameters and the reference state, it is possible that refinement of the model and/or enrichment of the dataset would make it a viable option.

The need for an estimate of the LV pressure curve for active estimation means that further validation of the employed scaled normalised data would be beneficial. While the curve in Russell et al. (2012) was shown to be similar for patients with a number of different heart conditions (and consistent between diseased and healthy canines), its application to personalised models of healthy volunteers is yet to be fully verified. Any errors in the pressure will translate linearly into errors in all parameter estimates due to their linear dependence prescribed by the model.

Experimental validation of these results would be an important step in ensuring that model-based estimates and predictions can be relied upon in clinical practice.

## Conclusions

The proposed pipeline for personalisation of the full-cycle LV mechanics model produced unique passive and active parameters for all of the in silico and in vivo datasets tested. Moreover, the behaviour of the objective functions over the respective parameter spaces implied that an efficient optimisation or filtering method can be successfully used to find the patient-specific parameters of the model. The choice of the $L_2(\varOmega _0)$-norm of displacement error as the objective function in parameter estimation allowed us to take advantage of the rich dataset extracted from 3D tagged MR images. In the in silico tests, the presence of random noise of up to 20 % and typical processing errors caused a moderate deterioration of model fidelity, without a significant effect on the objective function behaviour. The associated errors in parameter estimates were within the data noise/error level. In vivo estimation results showed good agreement with global measurements, such as the P–V loop and the overall motion of the ventricle.

## Figures and Tables

**Fig. 12:**
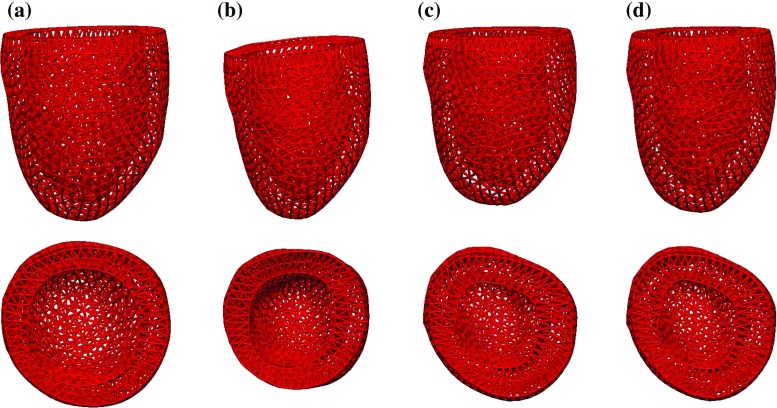
Reference meshes obtained by deflation of the end-diastolic mesh using the passive parameter ratios **c** $\gamma =0.05$ and **d** $\gamma =1$ compared to **a** the end-diastolic mesh and **b** the end-systolic mesh used as the reference state in the main body of the paper

**Fig. 13:**
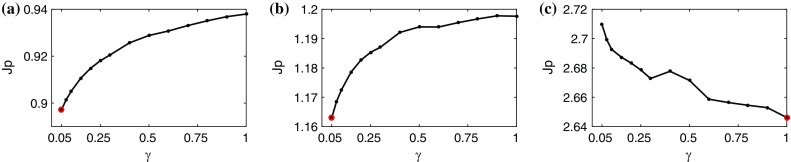
Variation of the modified objective function $\hat{{\mathcal {J}}}_p$ over the space of the passive parameter ratio based on estimated reference states for each ratio. The minimum is shown as a *circle*. The figures show the functional including **a** all diastolic frames starting at end systole, **b** all diastolic frames starting 3 frames past end systole, **c** only the end-diastolic frame

**Fig. 14:**
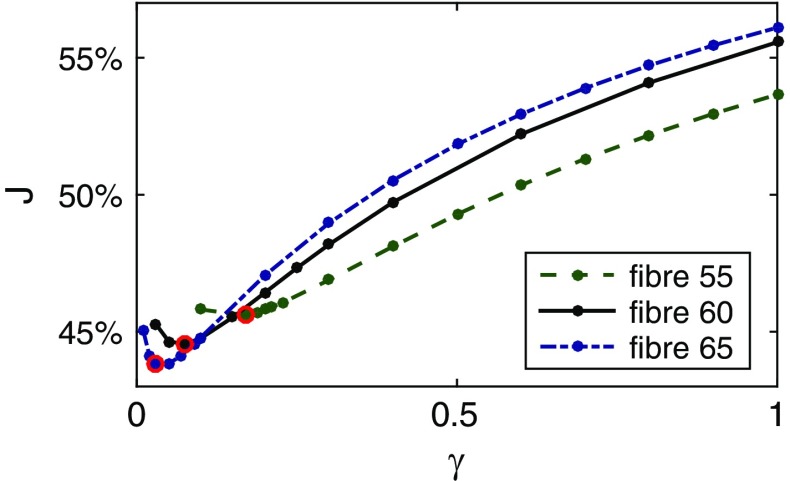
Effect of changing fibre distributions on passive estimation for in vivo case **V**

**Fig. 15:**
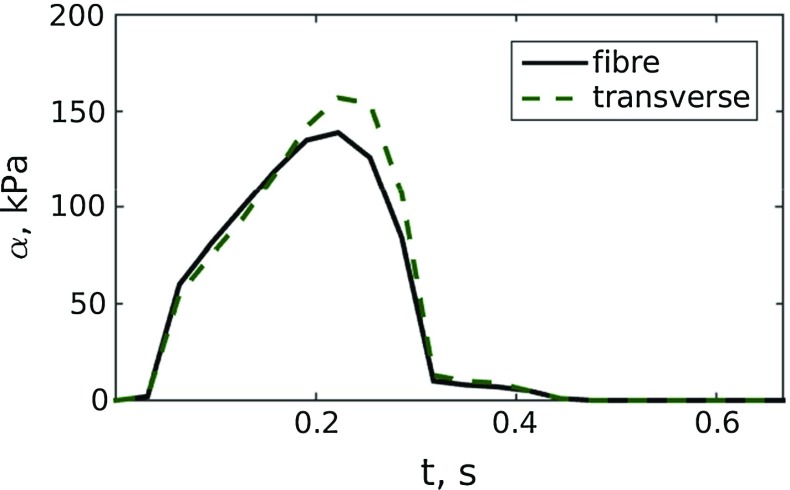
Estimated active tension using fibre-only activation, and fibre and transverse activation for case **V**

## Appendix 1: Joint estimation of the reference state and the passive parameters

Since the cardiac muscle is still activated at end systole, the approximation of the reference state by the end-systolic state is known to be inaccurate. We have therefore investigated the potential for estimating the unloaded state of the myocardium along with the passive parameters based on the data extracted from 3D tagged images, and the estimated reference volume and end-diastolic pressure for the in vivo case.

The estimation process was amended as follows:

1. Reference volume was estimated from the end-diastolic volume of the mesh and the end-diastolic pressure estimate based on (Klotz et al. 2006):
  $$\begin{aligned} V_{\text {ref}} = V_0 (0.6 - 0.006 \lambda _0) \approx 64\,\hbox {ml}. \end{aligned}$$
2. A deflation process consistent with the forward passive inflation model was carried out for a range of passive parameter ratios $\gamma \in [0.05,1]$ starting from the end-diastolic mesh and ending at the reference volume $V_{\text {ref}}$. The base of the ventricle was only restricted to allow no translation of the centre point of the base, no longitudinal motion and no rotation in the base plane. These conditions ensured wall thickening at the base consistent with the rest of the myocardium, and that non-physiological stresses at the base were not introduced post-inflation to end diastole. The reference states produced using limiting values of the $\gamma$ range are shown in Fig. 12 alongside the end-diastolic and the end-systolic meshes. Generally speaking, reference meshes got thinner and longer as $\gamma$ grew.
3. A sweep of inflation simulations over $\gamma \in [0.05,1]$ was performed as described in Sect. 3.2.1, each starting with the respective estimated reference state.
4. A modified objective function for computing the relative total displacement error was minimised to find the optimal passive parameter ratio.

The standard objective function ${\mathcal {J}}_p$ (9) was based on displacements to reference state, which would lead to inconsistency in a test where the reference state varied with $\gamma$. A modification was introduced, with displacements to end-diastolic state ${\varvec{x}}_0^{\text {ref}}$ serving as a consistent denominator, and the errors in the position of mesh points corresponding to a given parameter ratio ${\varvec{x}}_n^{\gamma }$ constituting the total error:

$$\begin{aligned} \hat{{\mathcal {J}}}_p[\gamma ] = \left( \frac{\displaystyle \sum _n \Vert {\varvec{x}}_n^{\gamma } - {\varvec{x}}_n^{\text {ref}}\Vert ^2}{\displaystyle \sum _n \Vert {\varvec{x}}_0^{\text {ref}} - {\varvec{x}}_n^{\text {ref}}\Vert ^2}\right) ^{1/2} \rightarrow \min _{\gamma }. \end{aligned}$$

The variation of the modified objective function over $\gamma$ is illustrated in Fig. 13. Since the myocardium experiences residual active tension in the end-systolic frame, and possibly a few frames after that, we considered different starting times for computing the total displacement error in the passive myocardium.^10^

The objective function $\hat{{\mathcal {J}}}_p$ does not have a minimum, whether all frames starting from end systole are included in the total or several frames showing the myocardium potentially experiencing residual active tension are excluded. The identifiability of passive parameters is therefore compromised by the inclusion of reference state estimation in the pipeline. The results might be improved if model fidelity was increased, e. g. with inclusion of a boundary traction on the region of attachment of the two ventricles. However, the coupling between the passive parameters and the reference state does not permit their simultaneous estimation from the available data.

## Appendix 2: Model adjustments

As discussed in Sect. 4, it is desirable to improve model fidelity for the pipeline to produce accurate parameter estimates. In this section, we look at the effect of two specific modelling choices made above, namely the distribution of the fibre orientations and the active constitutive law.

### Fibre distribution

In this section, we considered the effect of fibre angle distribution in the proposed pipeline.

Three sets of fibres were generated for the in vivo case **V**, all linearly varying through the wall, with angles ranging between $55^{\circ }$ and $-55^{\circ }, 60^{\circ }$ and $-60^{\circ }$, and $65^{\circ }$ and $-65^{\circ }$ going from the endo- to the epicardial surface.

The estimation procedure was carried out as described in Sect. 2.4. The variation of the passive objective function for the three tests is shown in Fig. 14.

The passive parameter values scaled by end-diastolic pressures are shown in Table 6.

**Table 6:** Estimated passive parameter values and active tension characteristics for different fibre distributions for in vivo case **V**

Endo fibre angle	$55^{\circ }$	$60^{\circ }$	$65^{\circ }$
$\gamma$	0.17	0.07	0.03
*a* (kPa)	0.476	0.274	0.142
$a_f$ (kPa)	2.800	3.909	4.740
$\alpha _{\max }$ (kPa)	142	139	138
$\hat{t}_{\max }$ (ms)	0.23	0.23	0.23
$\min _t {\mathcal {J}}_a$ (%)	24	24	24
$\min _t {\mathcal {J}}_a$ (%)	40	35	33

We observed that the total displacement error ${\mathcal {J}}_p$ at the minimum reduced as the fibre angles were increased, as did the optimal value of the passive ratio. Increasing the fibre angles in diastole increased dilation of the LV in short axis and reduces elongation. The reduced ratio $\gamma$ balanced this by reducing dilation and increasing elongation. Identifiability of parameters was not dramatically affected, and the change in error at the minimum of ${\mathcal {J}}_p$ was $<$2 %.

The estimated active tension profile and identifiability in ${\mathcal {J}}_a$ were not significantly affected by the chosen fibre distribution. The characteristics of the optimal $\alpha (t)$ and the associated ${\mathcal {J}}_a$ are shown in Table 6, and we also note that the highest difference between the estimated tensions for the different fibre orientations was 3 kPa at peak activation. A personalised fibre distribution, such as that based on diffusion tensor MRI, might have a stronger effect on model fidelity; however, this type of data was not available in the current datasets.

### Transverse activation

The simple active model used in this work and a lot of the more complex models assume that active systolic stress is developed mostly in the direction of the fibres. However, it has been suggested through experiment and computational modelling (Usyk et al. 2001, 2002) that activation at the level of $\sim$30 % of fibre stress occurs in the directions transverse to the fibres. We have carried out a test for case **V** in order to assess whether the inclusion of the transverse components in the active constitutive law has the potential to improve model fidelity. As a first approximation, the tension in each of the transverse directions was taken to equal 30 % of the tension in the fibre direction. A comparison of the estimated active tension scaling $\alpha (t)$ obtained using the two methods is shown in Fig. 15.

The range of total errors ${\mathcal {J}}_a$ in systole is reduced at the minimum over $\alpha$ from 24–35 to 18–26 %. The identifiability using the objective ${\mathcal {J}}_a$ remains unaffected, and identification of a physiological active tension based on the displacement component of the active functional is still impossible.

## Footnotes

1 http://www.doc.ic.ac.uk/~dr/software/.

2 For the purposes of the present study, flow through mitral plane would have been sufficient.

3 Computational Geometry Algorithms Library, http://www.cgal.org.

4 http://www-sop.inria.fr/asclepios/software/CardioViz3D.

5 http://www.gyrotools.com/products/gt-flow.html.

6 http://www.centrondiagnostics.com.

7 Near the minimum, the variation of ${\mathcal {J}}_p$ is slower in the $a_f$ direction than in the *a* direction (Hadjicharalambous et al. 2014b), and it is therefore preferable to fix $a_f$ when estimating the passive parameter ratio.

8 http://cheart.co.uk/.

9 The structured hexahedral mesh consisted of two conforming layers: the majority of the myocardium was subdivided into 8 elements circumferentially, 3 in the long-axis direction and 2 transmurally, and the apical patch subdivided into $2\times 2\times 2$ elements (see Fig. 1a). This resolution was sufficient to characterise objective function behaviour, as observed in Hadjicharalambous et al. (2014b).

## References

[CR1] Asner L, Hadjicharalambous M, Lee J, Nordsletten D (2015) STACOM Challenge: simulating left ventricular mechanics in the canine heart. In: Statistical atlases and computational models of the heart-imaging and modelling challenges, vol 8896. Springer, pp 123–134

[CR2] Augenstein KF, Cowan BR, LeGrice IJ, Nielsen PMF, Young AA (2005). Method and apparatus for soft tissue material parameter estimation using tissue tagged magnetic resonance imaging. J Biomech Eng.

[CR3] Babuska I (1973). The finite element method with Lagrangian multipliers. Numer Math.

[CR4] Bonet J, Wood R (2008). Nonlinear continuum mechanics for finite element analysis.

[CR5] Brenner SC, Scott R (2008). The mathematical theory of finite element methods.

[CR6] Brett SE, Guilcher A, Clapp B, Chowienczyk P (2012). Estimating central systolic blood pressure during oscillometric determination of blood pressure. Blood Press Monit.

[CR7] Buyens F, Jolivet O, De Cesare A, Bittoun J, Herment A, Tasu J-P, Mousseaux E (2005). Calculation of left ventricle relative pressure distribution in MRI using acceleration data. Magn Reson Med.

[CR8] Camara O., Mansi T., Pop M., Rhode K., Sermesant M., Young A. (2015) Statistical atlases and computational models of the heart-imaging and modelling challenges: 5th international workshop, STACOM 2014, Held in Conjunction with MICCAI 2014, Boston, MA, USA, September 18, 2014, Revised Selected Papers, vol 8896. Springer

[CR9] Casoni E, Jérusalem A, Samaniego C, Eguzkitza B, Lafortune P, Tjahjanto D, Sáez X, Houzeaux G, Vázquez M (2014) Alya: computational solid mechanics for supercomputers. Archives of Computational Methods in Engineering, pp 1–20

[CR10] Chabiniok R, Moireau P, Lesault P-F, Rahmouni A, Deux J-F, Chapelle D (2012). Estimation of tissue contractility from cardiac cine-MRI using a biomechanical heart model. Biomech Model Mechanobiol.

[CR11] Chandrashekara R, Mohiaddin RH, Rueckert D (2004). Analysis of 3-D myocardial motion in tagged MR images using nonrigid image registration. IEEE Trans Med Imaging.

[CR12] Chapelle D, Le Tallec P, Moireau P, Sorine M (2012). Energy-preserving muscle tissue model: formulation and compatible discretizations. Int J Multiscale Comput Eng.

[CR13] Chen C-H, Nevo E, Fetics B, Pak PH, Yin FC, Maughan WL, Kass DA (1997). Estimation of central aortic pressure waveform by mathematical transformation of radial tonometry pressure validation of generalized transfer function. Circulation.

[CR14] Costa KD, Holmes JW, McCulloch AD (2001). Modelling cardiac mechanical properties in three dimensions. Philos Trans R Soc A Math Phys Eng Sci.

[CR15] Dokos S, Smaill BH, Young AA, LeGrice IJ (2002). Shear properties of passive ventricular myocardium. Am J Physiol Heart Circul Physiol.

[CR16] Donati F, Figueroa CA, Smith NP, Lamata P, Nordsletten DA (2015) Non-invasive pressure difference estimation from PC-MRI using the work-energy equation. Med Image Anal 10.1016/j.media.2015.08.012 PMC4686008 26409245

[CR17] Ebbers T, Wigström L, Bolger AF, Engvall J, Karlsson M (2001). Estimation of relative cardiovascular pressures using time-resolved three-dimensional phase contrast mri. Magn Reson Med.

[CR18] Gao H, Carrick D, Berry C, Luo XY (2015) Parameter estimation of the Holzapfel–Ogden law for healthy myocardium. J Eng Math 1–18 10.1007/s10665-014-9740-3 PMC4662962 26663931

[CR19] Ge L, Ratcliffe M (2009). The use of computational flow modeling (CFD) to determine the effect of left ventricular shape on blood flow in the left ventricle. Ann Thorac Surg.

[CR20] Göktepe S, Acharya S, Wong J, Kuhl E (2011). Computational modeling of passive myocardium. Int J Numer Methods Biomed Eng.

[CR21] Guccione JM, Costa KD, McCulloch AD (1995). Finite element stress analysis of left ventricular mechanics in the beating dog heart. J Biomech.

[CR22] Gurev V, Pathmanathan P, Fattebert J-L, Wen H-F, Magerlein J, Gray RA, Richards DF, Rice JJ (2015) A high-resolution computational model of the deforming human heart. Biomech Model Mechanobiol 1–21 10.1007/s10237-014-0639-8 25567753

[CR23] Hadjicharalambous M, Lee J, Smith NP, Nordsletten DA (2014a) A displacement-based finite element formulation for incompressible and nearly-incompressible cardiac mechanics. Comput Methods Appl Mech Eng 274:213–236 10.1016/j.cma.2014.02.009 PMC4026127 25187672

[CR24] Hadjicharalambous M, Chabiniok R, Asner L, Sammut E, Wong J, Carr-White G, Lee J, Razavi R, Smith N, Nordsletten D (2014b) Analysis of passive cardiac constitutive laws for parameter estimation using 3D tagged MRI. Biomech Model Mechanobiol 1–22 10.1007/s10237-014-0638-9 PMC4490188 25510227

[CR25] Holzapfel GA, Ogden RW (2009). Constitutive modelling of passive myocardium: a structurally based framework for material characterization. Philos Trans Ser A Math Phys Eng Sci.

[CR26] Imperiale A., Chabiniok R., Moireau P., Chapelle D.(2011) Constitutive Parameter Estimation Methodology Using Tagged-MRI Data. In: *Proceedings of Functional Imaging and Modelling of the Heart 2011*. Springer, pp 409–417

[CR27] Kerckhoffs RCP, Bovendeerd P, Prinzen F, Smits K, Arts T (2003). Intra-and interventricular asynchrony of electromechanics in the ventricularly paced heart. J Eng Math.

[CR28] Kerckhoffs RCP, Neal ML, Gu Q, Bassingthwaighte JB, Omens JH, McCulloch AD (2007). Coupling of a 3D finite element model of cardiac ventricular mechanics to lumped systems models of the systemic and pulmonic circulation. Ann Biomed Eng.

[CR29] Klotz S, Hay I, Dickstein ML, Yi G-H, Wang J, Maurer MS, Kass Da, Burkhoff D (2006). Single-beat estimation of end-diastolic pressure–volume relationship: a novel method with potential for noninvasive application. Am J Physiol Heart Circul Physiol.

[CR30] Krishnamurthy A, Villongco CT, Chuang J, Frank LR, Nigam V, Belezzuoli E, Stark P, Krummen DE, Narayan S, Omens JH, McCulloch AD, Kerckhoffs RCP (2013). Patient-specific models of cardiac biomechanics. J Comput Phys.

[CR31] Lafortune P, Aris R, Vázquez M, Houzeaux G (2012). Coupled electromechanical model of the heart: parallel finite element formulation. Int J Numer Methods Biomed Eng.

[CR32] Marchesseau S, Delingette H, Sermesant M, Cabrera-Lozoya R, Tobon-Gomez C, Moireau P, Figueras i Ventura RM, Lekadir K, Hernandez A, Garreau M, Donal E, Leclercq C, Duckett SG, Rhode KS, Rinaldi CA, Frangi AF, Razavi RS, Chapelle D, Ayache N (2013). Personalization of a cardiac electromechanical model using reduced order unscented Kalman filtering from regional volumes. Med Image Anal.

[CR33] Markl M, Kilner PJ, Ebbers T (2011). Comprehensive 4D velocity mapping of the heart and great vessels by cardiovascular magnetic resonance. J Cardiovasc Magn Reson.

[CR34] McCormick M, Nordsletten D, Kay D, Smith N (2011) Modelling left ventricular function under assist device support. Int J Numer Methods Biomed Eng 27(7):1073–1095

[CR35] McCormick M, Nordsletten DA, Kay D, Smith NP (2013) Simulating left ventricular fluid—solid mechanics through the cardiac cycle under LVAD support. J Comput Phys 244:80–96

[CR36] Moireau P, Chapelle D, Tallec PL (2008). Joint state and parameter estimation for distributed mechanical systems. Comput Methods Appl Mech Eng.

[CR37] Mojsejenko D, McGarvey JR, Dorsey SM, Gorman JH, Burdick JA, Pilla JJ, Gorman RC, Wenk JF (2014) Estimating passive mechanical properties in a myocardial infarction using MRI and finite element simulations. Biomech Model Mechanobiol 1–15 10.1007/s10237-014-0627-z PMC4398581 25315521

[CR38] Nagler A, Bertoglio C, Gee M, Wall W (2013) Personalization of cardiac fiber orientations from image data using the unscented Kalman filter. In: *Functional imaging and modeling of the heart*. Springer, pp 132–140

[CR39] Nagueh SF, Middleton KJ, Kopelen HA, Zoghbi WA, Quin MA (1997). Doppler tissue imaging: a noninvasive technique for evaluation of left ventricular relaxation and estimation of filling pressures. J Am College Cardiol.

[CR40] Nagueh SF, Appleton CP, Gillebert TC, Marino PN, Oh JK, Smiseth OA, Waggoner AD, Flachskampf FA, Pellikka PA, Evangelisa A (2009). Recommendations for the evaluation of left ventricular diastolic function by echocardiography. Eur J Echocardiogr.

[CR41] Nash MP, Hunter PJ (2001). Computational mechanics of the heart. J Elast.

[CR42] Neal ML, Kerckhoffs RCP (2010). Current progress in patient-specific modeling. Briefings Bioinf.

[CR43] Niederer SA, Plank G, Chinchapatnam P, Ginks M, Lamata P, Rhode KS, Rinaldi CA, Razavi R, Smith NP (2011). Length-dependent tension in the failing heart and the efficacy of cardiac resynchronization therapy. Cardiovasc Res.

[CR44] Rohmer D., Sitek A., Gullberg G.T. (2006) Reconstruction and visualization of fiber and sheet structure with regularized tensor diffusion MRI in the human heart. Lawrence Berkeley National Laboratory Publication. LBNL-60277

[CR45] Rueckert D, Sonoda LI, Hayes C, Hill DL, Leach MO, Hawkes DJ (1999). Nonrigid registration using free-form deformations: application to breast MR images. IEEE Trans Med Imaging.

[CR46] Russell K, Eriksen M, Aaberge L, Wilhelmsen N, Skulstad H, Remme EW, Haugaa KH, Opdahl A, Fjeld JG, Gjesdal O, Edvardsen T, Smiseth OA (2012). A novel clinical method for quantification of regional left ventricular pressurestrain loop area: A non-invasive index of myocardial work. Eur Heart J.

[CR47] Rutz AK, Ryf S, Plein S, Boesiger P, Kozerke S (2008). Accelerated whole-heart 3D CSPAMM for myocardial motion quantification. Magn Reson Med.

[CR48] Sainte-Marie J, Chapelle D, Cimrman R, Sorine M (2006). Modeling and estimation of the cardiac electromechanical activity. Comput Struct.

[CR49] Sermesant M, Chabiniok R, Chinchapatnam P, Mansi T, Billet F, Moireau P, Peyrat J, Wong K, Relan J, Rhode K, Ginks M, Lambiase P, Delingette H, Sorine M, Rinaldi C, Chapelle D, Razavi R, Ayache N (2012). Patient-specific electromechanical models of the heart for the prediction of pacing acute effects in CRT: A preliminary clinical validation. Med Image Anal.

[CR50] Shi W, Zhuang X, Wang H, Duckett SG, Luong DVN, Tobon-gomez C, Tung K, Edwards PJ, Rhode KS, Razavi RS, Ourselin S, Rueckert D (2012). A comprehensive cardiac motion estimation framework using both untagged and 3-D tagged MR images based on nonrigid registration. IEEE Trans Med Imaging.

[CR51] Shi W, Jantsch M, Aljabar P, Pizarro L, Bai W, Wang H, O’Regan D, Zhuang X, Rueckert D (2013). Temporal sparse free-form deformations. Med Image Anal.

[CR52] Sommer G, Schriefl AJ, Andrä M, Sacherer M, Viertler C, Wolinski H, Holzapfel GA (2015). Biomechanical properties and microstructure of human ventricular myocardium. Acta Biomater.

[CR53] Spotnitz HM (2000). Macro design, structure, and mechanics of the left ventricle. J Thorac Cardiovasc Surg.

[CR54] Stoeck CT, Kalinowska A, von Deuster C, Harmer J, Chan RW, Niemann M, Manka R, Atkinson D, Sosnovik DE, Mekkaoui C, Kozerke S (2014). Dual-phase cardiac diffusion tensor imaging with strain correction. PloS ONE.

[CR55] Streeter DD, Spotnitz HM, Patel DP, Ross J, Sonnenblick EH (1969). Fiber orientation in the canine left ventricle during diastole and systole. Circ Res.

[CR56] The CGAL Project. CGAL User and Reference Manual. CGAL Editorial Board, 4.6 edition, 2015. URL http://doc.cgal.org/4.6/Manual/packages.html

[CR57] Toussaint N., Mansi T., Delingette H., Ayache N., Sermesant M. (2008) An integrated platform for dynamic cardiac simulation and image processing: Application to personalised tetralogy of fallot simulation. In: Eurographics workshop on visual computing for biomedicine (VCBM), Delft, The Netherlands

[CR58] Toussaint N., Stoeck C.T., Schaeffter T., Kozerke S., Sermesant M., Batchelor P.G. (2013) *In vivo* human cardiac fibre architecture estimation using shape-based diffusion tensor processing. Medical Image Anal 1243–1255 10.1016/j.media.2013.02.008 23523287

[CR59] Tyszka JM, Laidlaw DH, Asa JW, Silverman JM (2000). Three-dimensional, time-resolved (4d) relative pressure mapping using magnetic resonance imaging. J Magn Reson Imaging.

[CR60] Uribe S, Muthurangu V, Boubertakh R, Schaeffter T, Razavi R, Hill DL, Hansen MS (2007). Whole-heart cine MRI using real-time respiratory self-gating. Magn Reson Med.

[CR61] Uribe S, Tangchaoren T, Parish V, Wolf I, Razavi R, Greil G, Schaeffter T (2008). Volumetric cardiac quantification by using 3D dual-phase whole-heart MR imaging. Radiology.

[CR62] Usman M, Atkinson D, Odille F, Kolbitsch C, Vaillant G, Schaeffter T, Batchelor PG, Prieto C (2013). Motion corrected compressed sensing for free-breathing dynamic cardiac MRI. Magn Reson Med.

[CR63] Usman M, Atkinson D, Heathfield E, Greil G, Schaeffter T, Prieto C (2015) Whole left ventricular functional assessment from two minutes free breathing multi-slice CINE acquisition. Phys Med Biol 60(7):N93–N107 10.1088/0031-9155/60/7/N93 25768044

[CR64] Usyk TP, Mazhari R, Mcculloch AD (2001) Effect of laminar orthotropic myofiber architecture on regional stress and strain in the canine left ventricle. J Elast 61:143–164

[CR65] Usyk TP, Legrice IJ, Mcculloch AD (2002). Computational model of three-dimensional cardiac electromechanics. Comput Vis Sci.

[CR66] Wang VY, Lam HI, Ennis DB, Cowan BR, Young AA, Nash MP (2009). Modelling passive diastolic mechanics with quantitative MRI of cardiac structure and function. Med Image Anal.

[CR67] Xi J, Lamata P, Shi W, Niederer S, Land S, Rueckert D, Duckett SG, Shetty AK, Rinaldi CA, Razavi R, Smith N (2011) An automatic data assimilation framework for patient-specific myocardial mechanical parameter estimation. Lect Notes Comput Sci 6666(2009):392–400

[CR68] Xi J, Lamata P, Niederer S, Land S, Shi W, Zhuang X, Ourselin S, Duckett SG, Shetty AK, Rinaldi CA, Rueckert D, Razavi R, Smith N (2013). The estimation of patient-specific cardiac diastolic functions from clinical measurements. Med Image Anal.

[CR69] Xi J, Shi W, Rueckert D, Razavi R, Smith NP, Lamata P (2014). Understanding the need of ventricular pressure for the estimation of diastolic biomarkers. Biomech Model Mechanobiol.

[CR70] Yacoub MH, Terracciano CM (2011). The Holy Grail of LVAD-induced reversal of severe chronic heart failure: the need for integration. Eur Heart J.

